# How to improve the diagnosis of neoplastic transformation of gastric hyperplastic polyps in the context of autoimmune gastritis?: A case report and lierature review

**DOI:** 10.1097/MD.0000000000032204

**Published:** 2022-12-02

**Authors:** Yunqi Xing, Haixiao Han, Yuxuan Wang, Zhongmei Sun, Linheng Wang, Dan Peng, Xiaoyuan Guo, Na Yao, Yali Yuan, Wenji Zhang, Tangyou Mao, Yuyue Liu

**Affiliations:** a Dongfang hospital, Beijing University of Chinese Medicine, Beijing, P.R China; b Graduate School, Beijing University of Chinese Medicine, Beijing, P.R. China.

**Keywords:** autoimmune gastritis, case report, diagnosis, gastric hyperplastic polyp, neoplastic transformation

## Abstract

Gastric hyperplastic polyp (GHP) commonly arises in the abnormal background mucosa, which makes it easy to be misdiagnosed and missed, and has a potential risk of malignant transformation over time. Here, we present a case of neoplastic transformation of GHP in a context of autoimmune gastritis (AIG).

**Patient concerns:**

In 2020, a 67-year-old woman was admitted for endoscopic review 6 years after gastric polyp resection, the histological diagnosis of gastric polyp was neoplastic transformation of GHP as before. The patient had undergone multiple polypectomies at the same part. Then histological examination revealed that partial epithelial hyperplasia and dysplasia, and the neoplastic areas were interlaced with normal mucosa.

**Diagnoses and interventions:**

We further found that the background diagnosis was AIG. These results supported the diagnosis of neoplastic transformation of GHP in a context of AIG. With the doubt of missed diagnose, we retrospectively analyzed the medical history in 2014, 2015 and 2016, confirmed the presence of AIG. Unfortunately, serological tests and special treatment were not performed.

**Outcomes:**

The correct diagnosis was eventually confirmed in 2020, which enables patients to receive normal treatment and monitoring, and avoids further deterioration of the disease.

**Lessons:**

The purpose of this case report is to increase clinical awareness of neoplastic transformation of GHP in a context of AIG, and hope promise for early diagnosis and treatment.

## 1. Introduction

Gastric hyperplastic polyp (GHP) is one of the most common polyps encountered in the stomach, especially in the antrum, and detected in 1.9% of 110,000 patients subjected to gastroscope.^[1]^ GHP occurs in all ages, but the high incidence age is 65 to 70 years old. Of which, 58% to 70.5% of patients with GHP are female,^[2]^ strongly indicating a gender difference in the occurrence and development of GHP. Morphologically, GHP ranges in size from a few millimeters to a few centimeters,^[3]^ leading to be misdiagnosed as tumor or cancer under endoscopy. Some of patients with GHP may present with dyspepsia, heartburn, abdominal pain, or anemia, but most of them are usually asymptomatic,^[4]^ which makes it difficult to be diagnosed in advance. GHP was considered to be a non-tumor lesion previously.^[5]^ However, an increasing number of studies^[6,7]^ have shown that GHP could be complicated with dysplasia and canceration, especially for polyps larger than 2 cm in diameter have a higher risk of neoplastic transformation.

In general, GHP occurs in the abnormal background mucosa, particularly in autoimmune gastritis (AIG), also known as type A gastritis.^[8]^ In the context of AIG, GHP is often multiple, large, pedunculate or subpedunculate, and easy to produce neoplastic transformation.^[3]^ Zhang et al^[9]^ reported that the incidence of neoplastic transformation of GHP in AIG is approximately 2.8%. In this paper, we report a case of GHP in a context of AIG misdiagnosed as adenomatous polyp. Fortunately, the correct diagnosis was eventually confirmed by comprehensive analysis of gastroscopy, pathology and serology. Therefore, when patients recurrent GHP in the body of gastric with a history of anemia, we should be alert to the possibility of AIG, especially repeated recurrence in the same position after polyp resection, formulate a reasonable gastroscopy and follow-up program to achieve early diagnosis and treatment, to avoid complications and neoplastic transformation.

## 2. Case presentation

**Figure 1.**
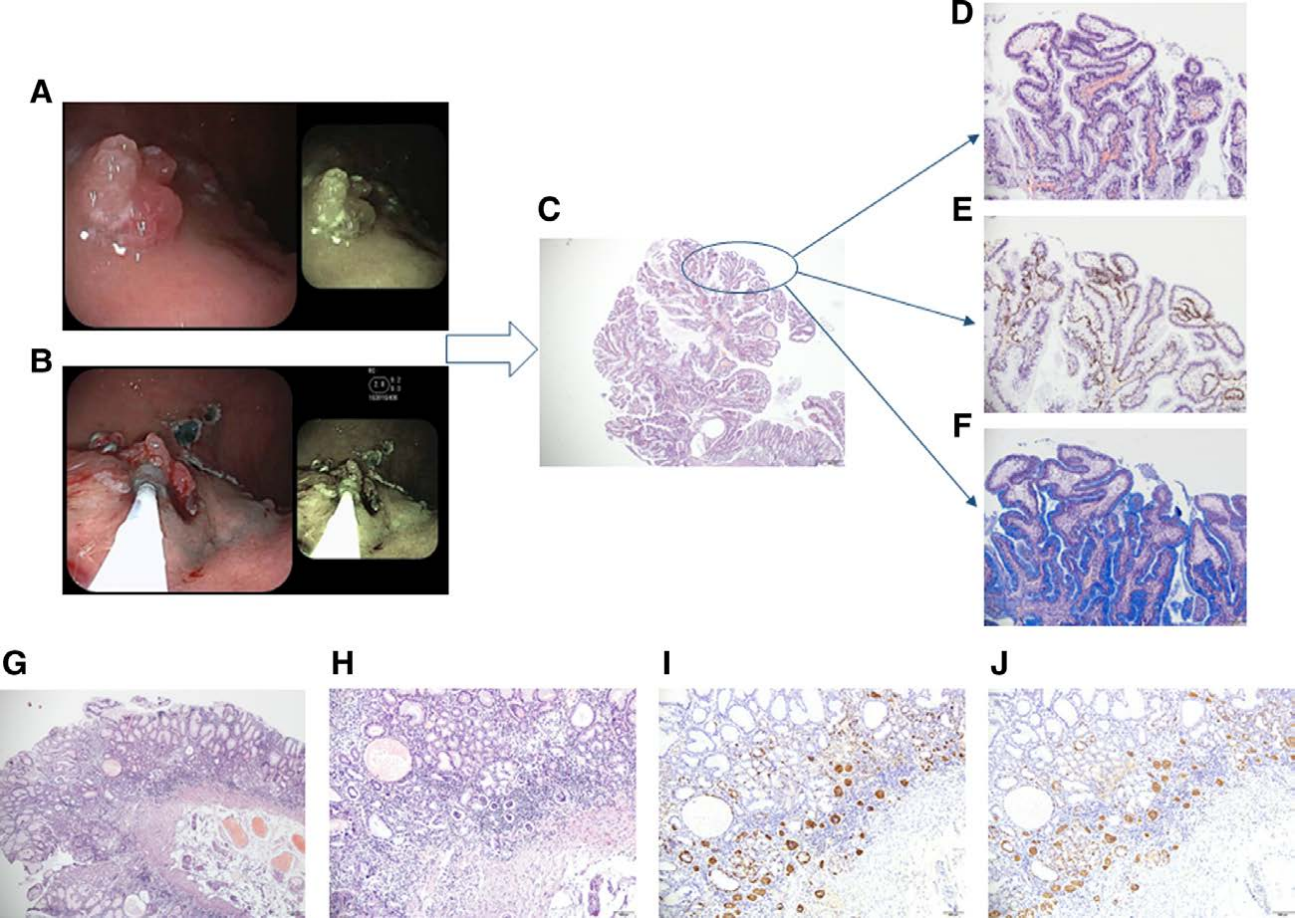
Endoscopic, histological images and examination of the background mucosa in 2020. (A) Multiple lobulated polyps with a size of 0.3 to 2.0 cm in the gastric body. (B) Endoscopic submucosal dissection. Electrotomy + electrocautery and endoscopic hemostasis were performed for the pedunculated polyp on the gastric body. (C) Low magnification image of the boxed area by hematoxylin and eosin (HE) staining (20X). (D) Partial epithelial hyperplasia and atypical hyperplasia (100X). (E) The neoplastic areas were interlaced with normal mucosa, and Ki-67 was positive in the whole layer of tumor area (100X). (F) The area of tumor change is characterized by a decrease in mucus-secreting goblet cells in the surface epithelium (100X). (G) Low magnification image of the boxed area by HE staining (40X). (H) Atrophy of inherent glands in gastric mucosa with intestinal metaplasia and pyloric gland metaplasia (100X). (I and J) Linear and nodular hyperplasia of ECL cells was revealed by chromogranin A and synaptophysin staining (100X).

In August 2020, a 67-year-old Chinese woman was admitted to our hospital for review 6 years after gastric polyp resection. Her symptoms included fatigue, poor appetite and lost 4 kg in a year, and she was accompanied by a 6-year history of iron deficiency anemia, which has been taking orally with iron and folic acid. The results from blood tests performed showed an iron deficiency anemia and decreased hemoglobin. No abnormalities were found in the Female Tumor Markers or Gastrointestinal Tumor Markers (Table 1). Upper endoscopy revealed multiple lobulated polyps with a size of 0.3 to 2.0 cm in the gastric body (Fig. 1A). Subsequently, electrotomy, electrocautery and endoscopic hemostasis were performed (Fig. 1B), and the diameter of the removed polyp specimen was 0.3 to 1.2 cm, which was gray and soft.

**Table 1:** Laboratory data in August 2020.

Laboratory findings	Value	Unit
White blood cells	4.07	×10^9^/μL
Red blood cells	4.2	×10^6^/μL
Mean corpuscular volume	83.3	fl
Hemoglobin	112*	g/dL
Hematocrit	35	%
Platelets	135	×10^4^/μL
Serum iron	7.23*	μmol/L
Total iron binding capacity	68	μmol/L
Ferritin	13.3	ng/mL
CA724	<1.500	U/mL
CEA	2.22	ng/mL
AFP	3.630	ng/mlL
Anti-parietal cell antibody	(+)	

* The patient’s value was below normal.

Interestingly, we discovered that the patient had undergone multiple polypectomies in the same part of the corpus, and was accompanied with anemia all the time. Therefore, we did histological examination and revealed that partial epithelial hyperplasia and dysplasia, and the neoplastic areas were interlaced with normal mucosa, as indicated by positive Ki-67 staining in the whole layer of tumor area and a decrease in mucus-secreting goblet cells in the surface epithelium. (Fig. 1C–F). These results supported the diagnosis of neoplastic transformation of GHP.

Through further clinical and pathological collaboration, we further found that the normal mucosa around polyps showed atrophy of inherent glands in gastric mucosa with intestinal metaplasia and pyloric gland metaplasia (Fig. 1G and H), and linear and nodular hyperplasia of ECL cells based on chromogranin A and synaptophysin staining (Fig. 1I and J). Meanwhile, the anti-parietal cell antibody was positive (Table 1). Collectively, these data demonstrated that the background diagnosis was AIG.

The patient had multiple surgeries with a long history of anemia, the onset of AIG, so what was the relationship between neoplastic polyps and AIG? Is there any possibility of misdiagnosis in this patient before? To address these questions, we reviewed the medical history.

**Figure 2.**
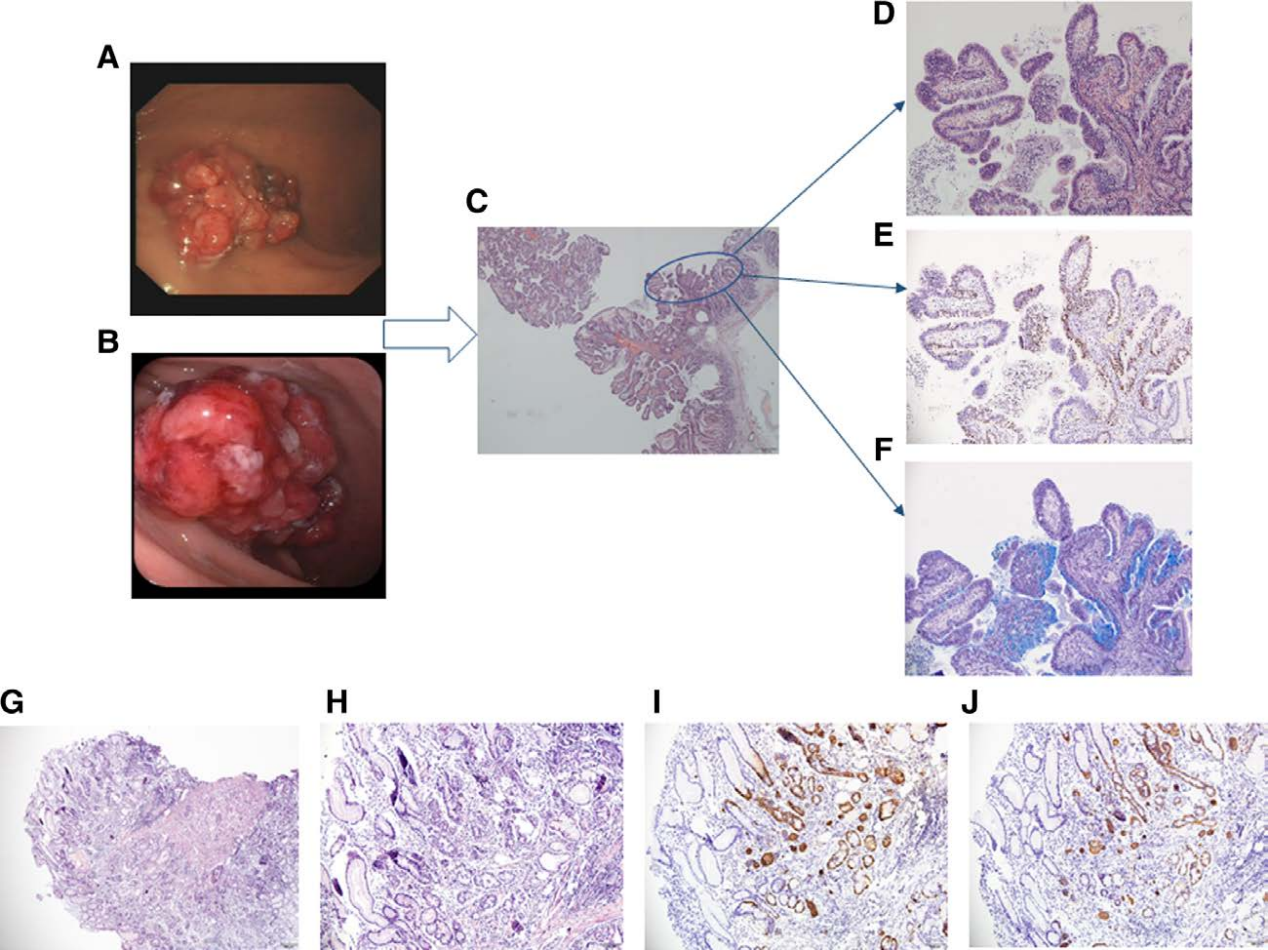
Endoscopic, histological findings and examination of the background mucosa in 2014. (A and B) 1.5 × 2.0 cm polyps in lobulated shape on the greater curvature of the gastric body, with scattered erosion on the surface. (C) Low magnification image of the boxed area by HE staining (20X). (D) Gastric hyperplastic polyp with neoplastic change in part of epithelium (100X). (E) Ki-67 staining of the border between the hyperplastic glands and the tumor glands. The tumor lesions were strongly positive for Ki-67 (+>15) (100X). (F) PAS staining showed a reduced mucus secretion in the tumor area (100X). (G)Low magnification image of the boxed area by HE staining (40X). (H) The original fundus glandular gastric mucosa by HE staining showed fewer inherent glands and pyloric gland metaplasia (100X). (I and J) Linear and nodular hyperplasia in ECL cells (100X).

In November 2014, the first visit of this patient was due to a physical examination. The results from positron emission tomography-computed tomography revealed gastric space occupation, as demonstrated by the vegetable pattern mass of the gastric wall on the greater curvature of the gastric body protrudes into the gastric cavity, and metabolism increases. Therefore, neoplastic lesions were considered. Upper endoscopy showed that there were 1.5 × 2.0 cm polyps in lobulated shape on the greater curvature of the gastric body, with scattered erosion on the surface (Fig. 2A and B). The pathological results showed that partial epithelial hyperplasia, and dysplasia, the tumor lesions were strongly positive for Ki-67 and PAS staining showed mucus secretion reduced, (gastric body) tubular adenomatous polyps were considered (Fig. 2C–F). The results of blood tests listed in Table 2 showed no obvious abnormality. Collectively, these results seemed to point to the diagnosis of neoplastic transformation of GHP.

**Table 2:** Laboratory data in November 2014.

Laboratory findings	Value	Unit
White blood cells	3.11	×10^9^/μL
Red blood cells	4.2	×10^6^/μL
Mean corpuscular volume	89.3	fl
Hemoglobin	118	g/dL
Hematocrit	37.5	%
Platelets	169	×10^4^/μl
Serum iron	8.50	μmol/L
Total iron binding capacity	48	μmol/L
Ferritin	17.1	ng/mL
CA724	1.340	U/mL
CEA	4.01	ng/mL
AFP	3.860	ng/mL

Retrospective analysis of the sections of the original fundus glandular gastric mucosa showed fewer inherent glands and pyloric gland metaplasia (Fig. 2G and H). Supplementary immunohistochemical staining confirmed the presence of specific linear and nodular hyperplasia of ECL cells in the gastric mucosa around adenomatous polyps, which was typical morphological evidence for AIG (Fig. 2I and J). Unfortunately, serological tests were not performed at that moment. Therefore, no special treatment was done except that the follow-up was informed 6 months later.

**Figure 3.**
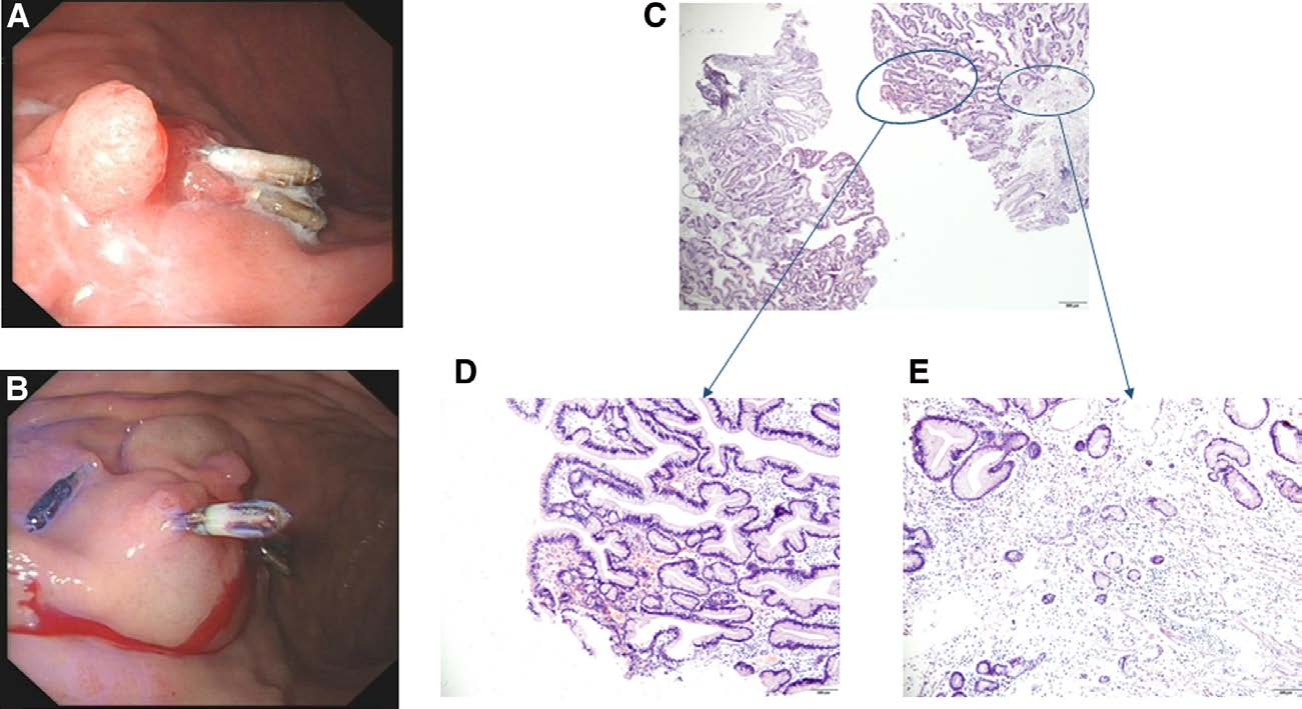
Endoscopic and histological findings in 2015. (A and B) an arty polypoid ridge with a size of about 0.4*0.4 cm. (C) Low magnification image of the boxed area (20X). (D) Gastric hyperplastic polyp with neoplastic change in part of epithelium (100X). (E) Nodular hyperplasia of a few ECL cells in surrounding background mucosa (100X).

In July 2015, upper endoscopy was repeated, and the results revealed that there were several titanium clips fixed on the mucosa in 2014, and an arty polypoid ridge with a size of about 0.4*0.4 cm in the same place. The mucosa around the surface of the hyperplasia was rough and less smooth, and polyp electrotomy was performed (Fig. 3A and B). Retrospective analysis of the biopsy pathology showed GHP with neoplastic change in part of epithelium and nodular hyperplasia of a few ECL cells in surrounding background mucosa (Fig. 3C–E). It suggested that the patient may have AIG, despite the absence of serological tests.

**Figure 4.**
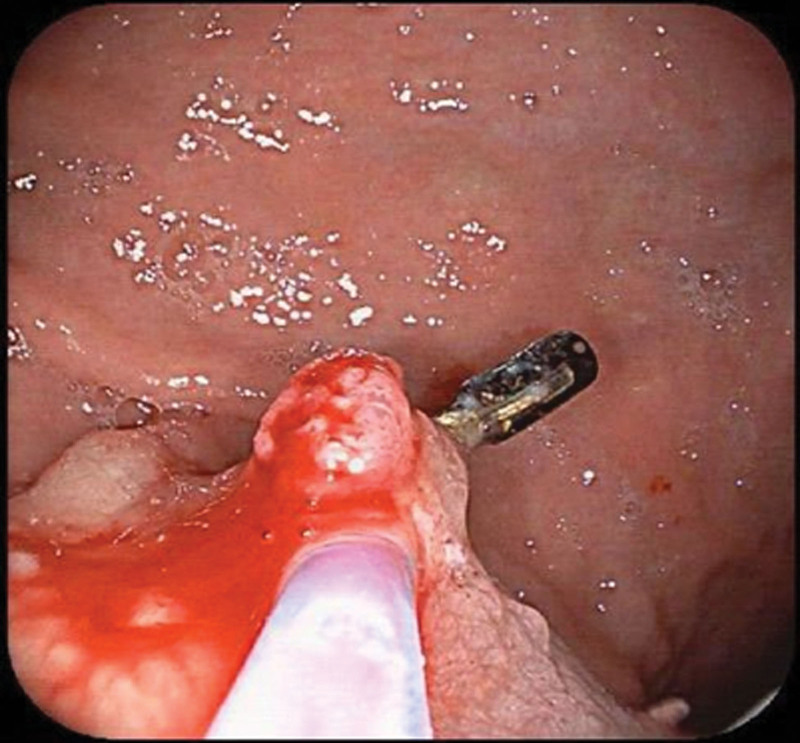
Endoscopic findings. The original titanium clip of the gastric body remained with small polyps around it.

In April 2016, the patient underwent upper endoscopy again and discovered the original titanium clip of the gastric body remained with small polyps around it, as shown in Figure 4. Later, removed together with the titanium clip. After that, the patient felt that his symptoms were well and did not undergo electronic gastroscopy review which is a very regrettable for the health of patient.

**Figure 5.**
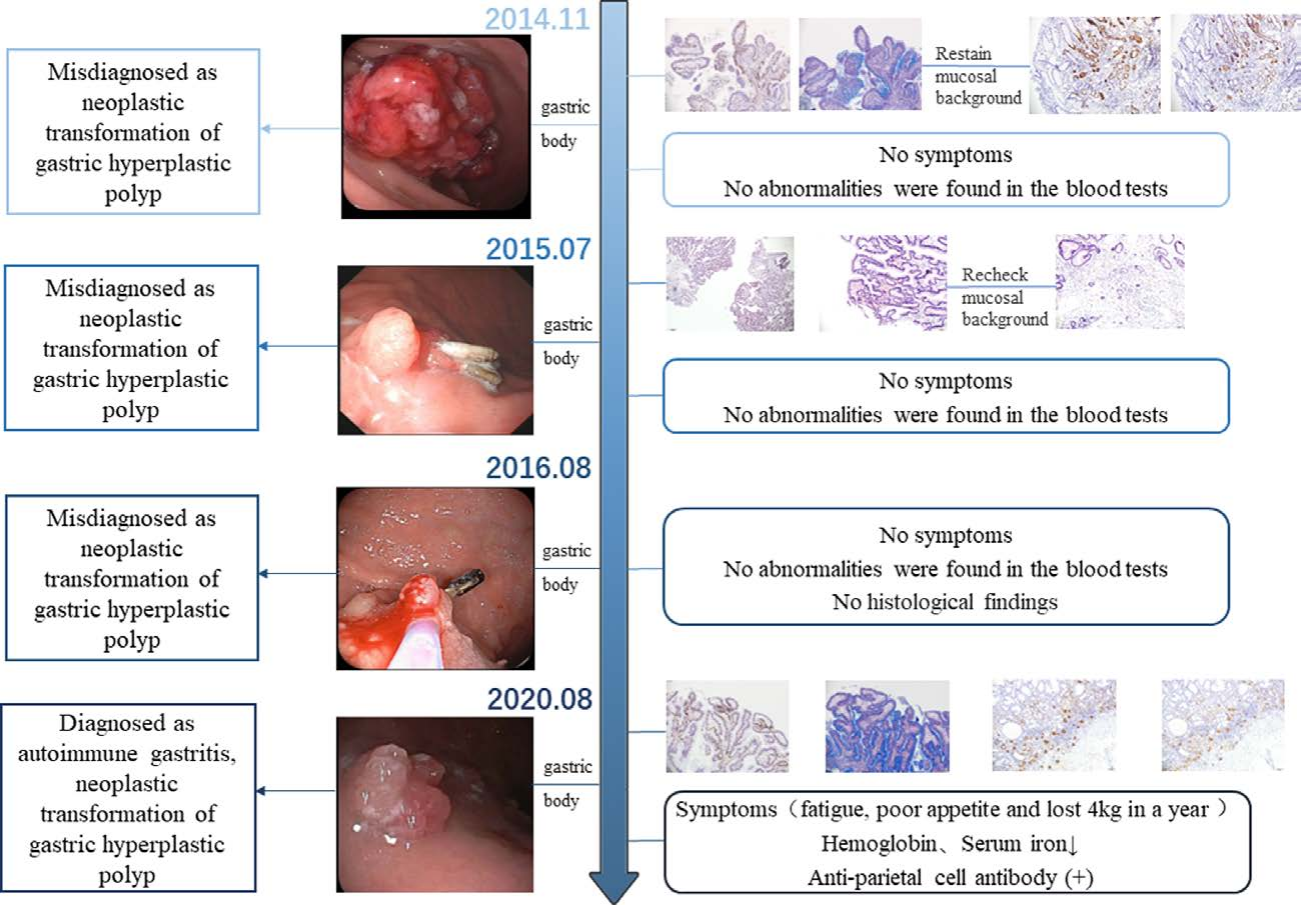
Case report timeline and process.

Therefore, the patient developed recurrent hyperplastic polyps with neoplastic changes on the same gastric mucosa, but AIG was missed diagnosis due to the neglect of the background. The whole clinical process and timeline of this patient is exhibited in Figure 5. Fortunately, the correct diagnosis was eventually confirmed by comprehensive analysis of gastroscopy, pathology and serology. Of course, many important information has also been ignored. For example, the patient was elderly female, the onset site was gastric corpus and polyps recurred in a short period after repeated resection, which were consistent with the epidemiological characteristics of AIG. Therefore, when patients recurrent GHP in the body of gastric with a history of anemia, we should be alert to the possibility of AIG, especially repeated recurrence in the same position after polyp resection.

## 3. Discussion and conclusion

GHP was considered to be a non-tumor lesion previously. An increasing number of studies have shown that GHP can be complicated with dysplasia and canceration, especially for polyps larger than 2 cm in diameter, the higher risk of neoplastic transformation. The reported average probability of neoplastic change of GHP ranges from 0.6% to 4.5%.^[10]^ Therefore, early diagnosis of GHP is of great clinical significance. At the present, pathologic diagnostic criteria of Nakamura for GHP with malignant transformation are as follows^[11]^: the coexistence of benign and malignant lesions in the same polyp; existence of sufficient evidence that malignant lesion was previously a benign polyp; sufficient cellular and structural dysplasia in the malignant area. Moreover, Ki-67 immunostaining and PAS staining also play an important role in the early diagnosis of neoplastic transformation of GHP. The expression of Ki-67 is able to assist in judging the development of tumor cells which can be used as a common indicator of cell proliferation activity. Shibahara et al^[12]^ suggested that examinations of Ki67 were important markers of malignant transformation of GHP, they conducted a study of GHP with malignant transformation and Ki-67 immunostaining showed positive in the carcinoma and dysplastic cells. PAS staining showed that mucin was diffusely positive in the epithelial surface cell nuclear and perinuclear membrane of normal gastric mucosa, some were homogeneous, some were diffusely stained. In contrast, the decrease or disappearance of PAS staining indicates the injury of normal mucosal epithelial cells/neoplastic lesions. Therefore, GHP has the possibility of malignant transformation and is easy to be misdiagnosed. Various methods need to be adopted for comprehensive diagnosis.

An increasing number of studies have shown that AIG can promote the neoplastic transformation of GHP.^[7,11]^ In general, GHP occurs in the abnormal background mucosa, particularly in AIG. Stimulated by high levels of gastrin, the association between GHP and AIG is well verified^[13]^ and the data demonstrated that 12% GHP related to AIG.^[14]^ The development of cancer is deemed to a multi-step process through hyperplasia-dysplasia-carcinoma sequence,^[15]^ and AIG confers an increased risk of intestinal-type gastric cancer.^[16]^ AIG is a chronic progressive inflammatory mediated by CD4 + T lymphocytes caused by autoantibodies attacking parietal cell, which can lead to atrophy and metaplasia of secretory acid glands in the corpus-fundus gastric mucosa, and involve digestive, blood, nerve and other systems. The high incidence of AIG occurs in older people, female and those with a history of autoimmune diseases. Moreover, Hershko et al^[17]^ reported that microcytic anemia which was present in 50% of patients with AIG. As the elapse of time, vitamin B12 is absorbed obstacle, and lead to megaloblastic anemia, even the effect of oral medicaments cannot be controlled and produce pernicious anemia. In AIG patients, gastric acid is reduced or even deficient due to atrophy of acid-secreting glands, resulting in reduced secretion of somatostatin, which is not sufficient to inhibit the secretion of gastrin by G cells, arising hypergastrinemia in response, causing hyperplasia of ECL in acid-secreting mucosa, occurring dysplasia as well as carcinoma.

In our patient, the patient developed recurrent hyperplastic polyps with neoplastic changes on the same gastric mucosa. The histological findings showed linear or nodular hyperplasia of enterochromaffin-like cell in the glandular of gastric mucosa, simultaneously, and the polyp morphology still maintained the morphological basis of GHP, gastric pits hyperplasia, elongation and expansion. However, some epithelial cells showed dysplasia, spreading from the proliferation zone to the surface. Ki-67 and PAS staining also supported the morphological interpretation results. All these results showed neoplastic transformation of GHP in a context of AIG. Unfortunately, we were not aware of possibility of AIG in our earlier work and draw the wrong conclusion.

From this case, we summarize some certain experiences. First of all, gastric polyp lesions ought to be considered according to different sites of distinct disease spectrum. Secondly, staying on highly vigilant when gastric antrum mucosa is normal, on the contrary existing abnormal or atrophic in gastric corpus mucosa, especially pay attention to track the basic information from patients, such as, age, gender and history of autoimmune diseases. Finally, during the endoscopy, the endoscopist ought to notice the mucosal status around polyps and gastric antrum mucosa and take samples not only from the biopsy site recommended by the Sydney system (5 biopsy samples should be obtained: two from the corpus, two from the antrum and one from the incisura angularis),^[18]^ but also take samples from the mucosa adjacent to the polyp. It may reduce missed diagnosis and misdiagnosis, particularly when the workload is heavy. Simultaneously, pathologists are supposed to focus on clinical information, cooperate closely with clinicians, discover diagnostic clues actively and follow up serological tests to assist diagnosis. Endoscopic monitoring should be considered in all patients with AIG, especially elderly patients.^[19]^ Once polyps are found in patients, in order to avoid the risk of cancer, endoscopic polypectomy should be considered in these GHP actively. To sum up, in order to make patients benefit more, clinicians, pathologists and endoscopist can study together and cooperate closely.

## Author contributions

YX collected patient information, analyzed results and wrote the first draft of the manuscript. HH collected information and performed the research. YW had pathological staining. ZS, LW, DP, XG, NY, YY, WZ did the upper gastrointestinal examination. TM supervised and interpreted the experimental data, critically revised the manuscript. YL designed the research, analyzed the data, and revised the manuscript critically. All authors read and approved the final manuscript.

**Conceptualization:** Yunqi Xing, Haixiao Han.

**Formal analysis:** Yunqi Xing, Yuxuan Wang, Zhongmei Sun, Linheng Wang, Dan Peng, Xiaoyuan Guo, Na Yao, Yali Yuan, Wenji Zhang.

**Methodology:** Haixiao Han.

**Supervision:** Tangyou Mao, Yuyue Liu.

**Writing – original draft:** Yunqi Xing.

**Writing – review & editing:** Tangyou Mao, Yuyue Liu.

## References

[1] Yu X Wang Z Wang L. Gastric hyperplastic polyps inversely associated with current *Helicobacter pylori* infection. Exp Ther Med. 2020;19:3143–9. 32256802 10.3892/etm.2020.8567 PMC7086145

[2] Park DY Lauwers GY. Gastric polyps: classification and management. Arch Pathol Lab Med. 2008;132:633–40. 18384215 10.5858/2008-132-633-GPCAM

[3] Ahn JY Son DH Choi KD. Neoplasms arising in large gastric hyperplastic polyps: endoscopic and pathologic features. Gastrointest Endosc. 2014;80:1005–13.e2. 24929480 10.1016/j.gie.2014.04.020

[4] Fujiwara-Tani R Okamoto A Katsuragawa H. BRAF mutation is associated with hyperplastic polyp-associated gastric cancer. Int J Mol Sci . 2021;22:12724. 34884530 10.3390/ijms222312724 PMC8657452

[5] Jung JT. Gastric polyps and protruding type gastric cancer. Clin Endosc. 2013;46:243–7. 23767034 10.5946/ce.2013.46.3.243 PMC3678061

[6] Zea-Iriarte WL Sekine I Itsuno M. Carcinoma in gastric hyperplastic polyps. A phenotypic study. Dig Dis Sci. 1996;41:377–86. 8601386 10.1007/BF02093832

[7] Daibo M Itabashi M Hirota T. Malignant transformation of gastric hyperplastic polyps. Am J Gastroenterol. 1987;82:1016–25. 3661508

[8] Karpińska-Kaczmarczyk K Lewandowska M Białek A. Gastric hyperplastic polyps coexisting with early gastric cancers, adenoma and neuroendocrine cell hyperplasia. Pol J Pathol. 2016;67:33–8. 27179272 10.5114/pjp.2016.59474

[9] Zhang H Nie X Song Z. Hyperplastic polyps arising in autoimmune metaplastic atrophic gastritis patients: is this a distinct clinicopathological entity? Scand J Gastroenterol. 2018;53:1186–93. 30353753 10.1080/00365521.2018.1514420

[10] Yamanaka K Miyatani H Yoshida Y. Malignant transformation of a gastric hyperplastic polyp in a context of Helicobacter pylori-negative autoimmune gastritis: a case report. BMC Gastroenterol. 2016;16:130. 27729029 10.1186/s12876-016-0537-x PMC5059938

[11] Han AR Sung CO Kim KM. The clinicopathological features of gastric hyperplastic polyps with neoplastic transformations: a suggestion of indication for endoscopic polypectomy. Gut Liver. 2009;3:271–5. 20431760 10.5009/gnl.2009.3.4.271 PMC2852734

[12] Shibahara K Haraguchi Y Sasaki I. A case of gastric hyperplastic polyp with malignant transformation. Hepatogastroenterology. 2005;52:319–21. 15783059

[13] João M Areia M Alves S. Gastric hyperplastic polyps: a benign entity? Analysis of recurrence and neoplastic transformation in a cohort study. GE Port J Gastroenterol. 2021;28:328–35. 34604464 10.1159/000514714 PMC8443946

[14] Hu H Zhang Q Chen G. Risk factors and clinical correlates of neoplastic transformation in gastric hyperplastic polyps in Chinese patients. Sci Rep. 2020;10:2582. 32054871 10.1038/s41598-020-58900-z PMC7018716

[15] Terada T. Malignant transformation of foveolar hyperplastic polyp of the stomach: a histopathological study. Med Oncol. 2011;28:941–4. 20458557 10.1007/s12032-010-9556-6

[16] Lahner E Esposito G Galli G. Atrophic gastritis and pre-malignant gastric lesions. Transl Gastrointest Cancer. 2015;4:272–81.

[17] Hershko C Ronson A Souroujon M. Variable hematologic presentation of autoimmune gastritis: age-related progression from iron deficiency to cobalamin depletion. Blood. 2006;107:1673–9. 16239424 10.1182/blood-2005-09-3534

[18] Dixon MF Genta RM Yardley JH. Classification and grading of gastritis. The updated Sydney system. International workshop on the histopathology of gastritis, Houston 1994. Am J Surg Pathol. 1996;20:1161–81. 8827022 10.1097/00000478-199610000-00001

[19] Mahmud N Stashek K Katona BW. The incidence of neoplasia in patients with autoimmune metaplastic atrophic gastritis: a renewed call for surveillance. Ann Gastroenterol. 2019;32:67–72. 30598594 10.20524/aog.2018.0325 PMC6302190

